# Supplementary material for: Formative Assessments Promote Procedural Learning and Engagement for Senior Pediatric Residents on Rotation in the Pediatric Emergency Department
Source: MedEdPORTAL. 2022 Jul 12;18:11265. doi: 10.15766/mep_2374-8265.11265 (PMC9273678; doi:10.15766/mep_2374-8265.11265)
Supplement: Supplementary file 1 — LP OSCE.docxLAC OSCE.docxPSIM Equipment List.docxPre-Post Questionnaire.docxFormative Feedback Report.docx [file mep_2374-8265.11265-s001.zip › A. LP OSCE.docx]

Appendix A: Infant Lumbar Puncture Informed Consent Formative Assessment

**Intro – To be read verbatim to the resident once the Zoom session begins:**

1. *Hi, thank you for your time. In the next 20 minutes or so, we will have two conversations around procedural consent. I will be the caregiver both times. You will be the doctor who will be performing the procedure on my child and therefor the task is to obtain informed consent to perform the procedure.*
2. *The setting of this procedure is the Pediatric Emergency Department (PED), which you have all worked in before.*
3. *Before we get started, please remember that formative assessments are SAFE! While it is possible you may have some anxiety right now, please know all this is in the vein of learning and practice.*
4. *To remind you, after this consent OSCE is completed, you will then do two observed procedural SIMs during your next week or so while on shift in the PED.*
5. *Also to remind you and hopefully reassure you, the primary goal of this work is to provide you, the resident, formative feedback on your approach to common pediatric procedures – from informed consent to performing the procedure to discharge planning.*
6. *You will receive a confidential packet of formative feedback on your performance once all formative assessments are completed.*
7. *And to be clear, formative feedback means this information will not go on any evaluations of yours and it will not be reported to your program directors. It is for you!*
8. *The best way for us to offer you valuable feedback is to video record these exercises to review your performance once completed.*
9. *To preserve anonymity, you can choose to hide your video on this ZOOM and we will only be recording your hands during the upcoming procedural SIMs.*
10. *Finally, this study protocol has been approved by your residency director, the Yale IRB and the Department of pediatrics Residency Research Oversight Committee.*
11. *OK, so do you have any questions?*
12. *If not, I will now press record.*
13. *At this point, I want to confirm you are OK with me pressing record?*
14. ***PRESS RECORD on ZOOM!!!!***

Case 1 – SP – Read this verbatim to the resident:

*-You are working in the Pediatric Emergency Department.*

*-You have already met Carl who is a 2-week-old baby boy who with fever.*

*-His caregiver’s name is Ms. Smith*

*-The RNs have placed an IV and sent blood and urine studies.*

*-You are responsible for the LP, which as you know, starts with discussing informed consent.*

*-We will start this scene under the assumption that you have already introduced yourself to the mom and examined Carl.*

*-Now you will tell the mom that you are here to discuss the LP.*

*-Any questions?*

*-If not, I will now get into “character.”*

Date:

All may be used

Primary Case Author:

Michael P. Goldman, MD

Secondary Case Author:

Marc A. Auerbach, MD MSCi

Standardized Patient Educator:

Ms. Joy Grabow

Name of Case:

Carl LP

Name of educational and or assessment activity:

Informed consent OSCE #1

Patient Name: Carl Smith

Chief Complaint: 2-week-old well appearing Male with a fever

Most likely Diagnosis and Differential with rationale from history and/or physical exam:

Fever in infant less than 21-days old, rule out sepsis and meningitis workup required. Case begins with need for informed consent for the LP.

Challenge questions:

*Is the LP 100% necessary?*

*How will you control the pain?*

*Can it cause paralysis?*

*Can we wait until my husband arrives?*

Domains:

- X Professionalism
- X Communication and Interpersonal skills
- Medical History
- Physical exam
- X Shared Decision Making
- X Patient Education
- Clinical Reasoning
- Documentation
- Handoff
- Presentation
- X Other: General procedural knowledge inclusive of risks/benefits, psychomotor techniques, anticipated pitfalls

Type and level of learner:

Senior Pediatric Residents

Case Objectives:

1. Communication / Interpersonal Skills; Professionalism; Procedural Knowledge:
   1. Demonstrate skills in obtaining informed consent for LP inclusive of the medical knowledge to justify the procedure, the risks vs. benefits tradeoff and ways one minimizes risk.
2. Patient / Caregiver Education; Shared Decision Making; Professionalism:
   1. Anticipate and address the common caregivers’ concerns and use appropriate language to reassure caregivers about the LP
3. Procedural Knowledge:
   1. Demonstrate knowledge and appropriate level of confidence in independently performing the LP

| SETTING: | Pediatric Emergency Department |
| --- | --- |
| PATIENT PROFILE: | |
| Age range | Young first time caregiver of 2-week-old baby boy |
| Religious/spiritual background | All may be used |
| Sex (e.g., male, female, intersex, transwoman, transman) | Female, though all may be used |
| Sexual Orientation (e.g., heterosexual, lesbian, gay, bisexual, pansexual, queer, asexual) | All may be used |
| Gender expression (e.g., man, woman, gender queer) | All may be used |
| Race/ethnicity: | All may be used |
| Physical description (e.g., BMI, height range) | All may be used |
| Physical limitations | None |
| Patient / Caregiver appearance (e.g., disheveled, hospital gown, business casual, casual) | Casual |
| Moulage + location (e.g., none, bruises, scars, body piercing, tattoos) | None |
| Affect (e.g., pleasant, cooperative) | Caregiver is non-obstructive but is appropriately concerned that her baby requires an invasive and potentially painful procedure. |
| Family group (e.g., who is family, who they live with) | Patient lives with mother (Ms. Smith) and father (Ms. Smith’s husband) who is on the way to the PED. |
| Education | Graduate degree outside of medicine |
| Level of health literacy | Mother is unfamiliar with the procedure but has baseline familiarity with medical procedures given her recent experience receiving an epidural block when she gave birth to Carl just 2 weeks ago. |
| Employment, if any - present and past, noting any current stresses | All may be used |
| Home/homeless - type of dwelling, number of stories, owned or rented | Domiciled, renting locally |
| Financial situation- any current stresses | All may be used |
| Insurance Status (e.g., un/under/insured, public/private, HMO/PPO) | Insured with public insurance |
| Habits (i.e., diet, exercise, caffeine, smoking, alcohol, drugs) | The caregiver does not display any concerning habits that would be relevant to this exercise. She is getting less sleep than typical but does not feel excessively irritable or sleep deprived. |
| Activities (i.e., hobbies, sports, clubs, friends) | Mom is a regular exerciser and getting the opportunity to resume this hobby. She is visiting with family and friends regularly while on parental leave. She is well supported by her husband and an extended family and friends network. |
| Typical day - what is the usual daily routine | Currently mom is on parental leave and she is assuming the primary caregiving role during the day as dad is recently back to work after a two-week parental leave. At night they switch off who wakes to attend and feed the baby. |

| CASE INFORMATION | |
| --- | --- |
| Chief Concern: | 2-week-old with fever |
| Additional Concerns: | Father not yet at bedside |
| THE PATIENT STORY: | My son Carl has a fever of 101 and I am very worried because he is only 2 weeks-old. I was told by the pediatrician that I need to bring my baby into the emergency room for any fever before 3 months old but I’m not sure why. He seems okay but is this something serious? Is it dangerous for babies to have a fever? |
| HISTORY OF PRESENT ILLNESS: | |
|  | |
| Onset (when; gradual or sudden) | Same day onset |
| Setting (what was going on or where was patient when symptoms first noticed?) | Carl woke up from a nap and felt warm to touch. |
| Duration (how long) | Just noticed an hour ago, called the pediatrician and they referred me to the Pediatric Emergency Department |
| Time relationships (frequency, constant or intermittent) | n/a |
| Location | n/a |
| Radiation | n/a |
| Quality | n/a |
| Amount | n/a |
| Aggravated by what | n/a |
| Relieved by what | n/a |
| Associated with what | Some mild congestion |
| Attitude (what does the patient think is the problem, and how does he/she feel about it) | The mother is concerned and reported to the Pediatric Emergency Department immediately as recommended by her pediatrician. |
| Overall course | Carl is stable but needs an extensive diagnostic workup |
| REVIEW OF SYSTEMS: Significant positives and negatives | |
|  | + = Fever, mild congestion |
|  | - = Respiratory distress, vomiting, diarrhea, rash, excessive fatigue, inability to tolerate feedings, changes in urine output |
|  |  |
|  | |
| Past medical history | Full term, healthy baby, thriving |
| Medication allergies (Name and reaction) | n/a |
| Environmental allergies (Name and reaction) | n/a |
| Illnesses | No prior illnesses |
| Vaccinations | Received hepatitis B #1 prior to leaving the newborn nursery |
| Surgeries | s/p uncomplicated circumcision prior to discharge from the newborn nursery |
| Accidents/ injuries/ trauma | None |
| Hospitalization | Uncomplicated newborn nursery stay |
|  | |
| Inclusive sexual and reproductive history | |
| Sexual practices  Sexual partners  Protection: Use of safer sex practices  Use of birth control if appropriate  Risk of intimate partner violence | Not applicable |
| Ob/GYN HISTORY | This is mom’s first pregnancy and first baby. She is healthy in her mid 20s and has no known medical problems or pre-natal risk factors inclusive of negative serologies for infectious diseases and Group B Strep colonization. |
| Medications | Carl takes a multivitamin |
| Immunizations | - Tetanus - Flu - X Hepatitis - Pneumovax - HPV - Other |
| Tobacco products:   - Cigarettes - Cigar - Pipe - Chew - E-cigarettes | - X Never - Past- year started/year quit - Current   - Quantity   - # of years |
| Alcohol   - Beer - Wine - Liquor - Other | - X Never - Past- year started/year quit - Current   - Quantity   - # of years |
| Drugs   - Weed - Cocaine - Heroin - Meth - Other - IV - Inhalants - Other | - X Never - Past- year started/year quit - Current   - Quantity - # of years |
| Diet (describe) | Breast and bottle fed, 45^th^ percentile on growth curve |
| Exercise (describe) | Not applicable |
| List any other important social history or information important to this case | Father is not currently w/ the mother and she would very much prefer to wait for his arrival before initiating the procedure |
| Family history |  |
| Mother, Father, Siblings, Grandparents, and other significant findings. | Not applicable |
|  |  |
| Physical Exam- | |
| PHYSICAL EXAM FINDINGS |  |
| 1. Written in layman’s terms | The baby does not need to be pictured in this exercise |
| 1. General appearance- affect, appearance, position of patient at opening (i.e. sitting, laying down, holding abdomen etc.) | WELL appearing |
| 1. Vital signs | 38.7 / 160 / 40 / 100% on RA / 100/60 |
| 1. Specific findings and affect | +Circumcision, flat fontanelle, normal cardiorespiratory exam |
| 1. Response to certain physical movements | Not applicable |
|  |  |
| DIAGNOSIS AND DIFFERENTIAL |  |
| Diagnosis with support from positive and negative history and PE findings | Sepsis, meningitis, urinary tract infection, bacteremia, virus |
| Differential with support from positive and negative history and PE findings | Virus |
|  |  |
| MANAGEMENT OR DIAGNOSTIC PLAN | Blood, urine studies, cerebrospinal fluid studies, antibiotics, hospital admission |
|  |  |
| PROFESSIONALISM ISSUES OR CHALLENGES: | Resident does not come off as confident or overconfident  Resident uses too much medical jargon  Resident does not check for understanding well  Resident does not explain the steps of the procedure well  Resident is disorganized  Resident refuses to wait for father to arrive prior to performing the Lumbar Puncture |
