# Supplementary material for: Formative Assessments Promote Procedural Learning and Engagement for Senior Pediatric Residents on Rotation in the Pediatric Emergency Department
Source: MedEdPORTAL. 2022 Jul 12;18:11265. doi: 10.15766/mep_2374-8265.11265 (PMC9273678; doi:10.15766/mep_2374-8265.11265)
Supplement: Supplementary file 1 — LP OSCE.docxLAC OSCE.docxPSIM Equipment List.docxPre-Post Questionnaire.docxFormative Feedback Report.docx [file mep_2374-8265.11265-s001.zip › B. LAC OSCE.docx]

Appendix B: Toddler Laceration Repair Informed Consent Formative Assessment

Case 2 – SP – Read this verbatim to the resident:

*-You are working in the Pediatric Emergency Department*

*-Jimmy, a 3-year old boy has a laceration above his left eyebrow.*

*-His mom’s name is Ms. Jones*

*-He ran into the edge of a door and other than this gash near his eyebrow, he is totally fine from a head, neck, facial or any other trauma perspective.*

*-The RNs have placed a cotton swab of LET on the wound 20 minutes ago.*

*-The laceration is 3cm in length, through the dermis and is mildly gaping.*

*-You are responsible for the Laceration Repair, which as you know, starts with discussing informed consent.*

*-We will start this scene under the assumption you have already examined Jimmy and you are now ready to discuss the laceration repair.*

*-Any questions?*

*-If not, I will now get into “character.”*

Date:

All may be used

Primary Case Author:

Michael P. Goldman, MD

Secondary Case Author:

Marc A. Auerbach, MD MSCi

Standardized Patient Educator:

Ms. Joy Grabow

Name of Case:

Toddler Forehead Laceration

Name of educational and or assessment activity:

Informed consent OSCE #2

Patient Name:

Jimmy Jones

Chief Complaint:

3yo boy with forehead laceration sustained 2 hours prior.

Most likely Diagnosis and Differential with rationale from history and/or physical exam:

3 cm laceration near eyebrow through the dermis and is mildly gaping.

Jimmy is displaying no signs or symptoms of head, neck, oral, facial or other significant traumas.

Challenge question:

Is a laceration repair completely necessary?

How will he possibly stay still?

Will it be painful?

Should this procedure be performed by a Plastic Surgeon?

Domains:

- X Professionalism
- X Communication and Interpersonal skills
- Medical History
- Physical exam
- X Shared Decision Making
- X Patient Education
- Clinical Reasoning
- Documentation
- Handoff
- Presentation
- X Other: General procedural knowledge inclusive of risks/benefits, psychomotor techniques, anticipated pitfalls

Type and level of learner:

Senior Pediatric Residents

Case Objectives:

1. Communication / Interpersonal Skills; Professionalism; Procedural Knowledge:
   1. Demonstrate skills in obtaining informed consent for Laceration repair inclusive of the medical knowledge to justify the procedure, the risks vs. benefits tradeoff and ways one minimizes risk.
2. Patient / Caregiver Education; Shared Decision Making; Professionalism:
   1. Anticipate and address the common caregivers’ concerns and use appropriate language to reassure caregivers about the Laceration repair.
3. Procedural Knowledge:
   1. Demonstrate knowledge and appropriate level of confidence in independently performing the Laceration repair.

| SETTING: outpatient, in patient, ED, home, nursing home, rehab, group etc. | Pediatric Emergency Department |
| --- | --- |
| PATIENT PROFILE: | |
| Age range | Young mother of toddler |
| Religious/spiritual background | All may be used |
| Sex (e.g., male, female, intersex, transwoman, transman) | Female, but all may be used |
| Sexual Orientation (e.g., heterosexual, lesbian, gay, bisexual, pansexual, queer, asexual) | All may be used |
| Gender expression (e.g., man, woman, gender queer) | All may be used |
| Race/ethnicity: | All may be used |
| Physical description (e.g., BMI, height range) | Ambulatory toddler with normal habitus |
| Physical limitations | None |
| Patient appearance (e.g., disheveled, hospital gown, business casual, casual) | Casually dressed caregiver |
| Moulage + location (e.g., none, bruises, scars, body piercing, tattoos) | None needed |
| Affect (e.g., pleasant, cooperative) | Caregiver is non-obstructive but is appropriately concerned that her toddler requires a potentially painful procedure and is nervous about how the providers will keep her child calm and still. |
| Family group (e.g., who is family, who they live with) | The mother is primary caregiver. Jimmy’s father lives in a different part of the country. Grandma is at home with mom and Jimmy. |
| Education | Graduate degree outside of medicine |
| Level of health literacy | Mother was unfamiliar with the specifics of the procedure, especially for toddlers, but has baseline familiarity with medical procedures and people needing to have their lacerations “sewed up.” |
| Employment, if any - present and past, noting any current stresses | All may be used |
| Home/homeless - type of dwelling, number of stories, owned or rented | Domiciled, renting |
| Financial situation- any current stresses | Not a stress |
| Insurance Status (e.g., un/under/insured, public/private, HMO/PPO) | Insured with public insurance |
| Habits (i.e., diet, exercise, caffeine, smoking, alcohol, drugs) | The caregiver does not display any concerning habits that would be relevant to this exercise. |
| Activities (i.e., hobbies, sports, clubs, friends) | Jimmy is in daycare and is an active toddler. |
| Typical day - what is the usual daily routine | Mom brings Jimmy to daycare and Grandma picks Jimmy up at the end of the day. Jimmy and Grandma have dinner and then mom is back home in time to put Jimmy to bed. |

| CASE INFORMATION | |
| --- | --- |
| Chief Concern: | 3-year-old son ran into edge of door and has a gash near his eyebrow |
| Additional Concerns: | Mother is most worried about how to safely keep him still and keep him calm during the procedure near his eye. |
|  | |
| THE PATIENT STORY: | My son ran into the edge of a door and cut his eyebrow open. He bled a lot initially but that has stopped. He seems normal aside from this big cut. I’m nervous he may need stitches. I don’t want him to be in pain and I’m worried that it will be a scar for the rest of his life. He does not do well with needles, so I don’t think that the procedure will go well and a mistake may be made if he moves. I’m especially concerned given the proximity to Jimmy’s eye. |
| HISTORY OF PRESENT ILLNESS: | |
|  | |
| Onset (when; gradual or sudden) | Same day, two hours ago now. |
| Setting (what was going on or where was patient when symptoms first noticed?) | Ran into edge of door at home |
| Duration (how long) | Hours ago |
| Time relationships (frequency, constant or intermittent) | n/a |
| Location | Forehead / eyebrow |
| Radiation | n/a |
| Quality | n/a |
| Amount | n/a |
| Aggravated by what | n/a |
| Relieved by what | n/a |
| Associated with what | n/a |
| Attitude (what does the patient think is the problem, and how does he/she feel about it) | Mother is concerned but is capable of both engaging in the conversation with the provider and keeping Jimmy calm at the same time. |
| Overall course |  |
| REVIEW OF SYSTEMS: Significant positives and negatives | |
|  | (+) = Gash above eyebrow |
|  | (-) = Loss of consciousness, altered mental status, oral bleeding, neck pain, vomiting |
|  | |
| Past medical history |  |
| Medication allergies (Name and reaction) | None |
| Environmental allergies (Name and reaction) | None |
| Illnesses | No major illnesses |
| Vaccinations | Up to date including Tetanus status |
| Surgeries | Uncomplicated circumcision in the newborn nursery |
| Accidents/ injuries/ trauma | Some minor bumps and bruises, but this is the first time he needed to be brought to the pediatric emergency department |
| Hospitalization | None |
|  | |
| Inclusive sexual and reproductive history | |
| Sexual practices  Sexual partners  Protection: Use of safer sex practices  Use of birth control if appropriate  Risk of intimate partner violence | Not applicable |
| Ob/GYN HISTORY | Not applicable |
| Medications | No daily medications |
| Immunizations | - X Tetanus - X Flu - X Hepatitis - X Pneumovax - X HPV - Other |
| Tobacco products:   - Cigarettes - Cigar - Pipe - Chew - E-cigarettes | - X Never - Past- year started/year quit - Current   - Quantity   - # of years |
| Alcohol   - Beer - Wine - Liquor - Other | - X Never - Past- year started/year quit - Current   - Quantity   - # of years |
| Drugs   - Weed - Cocaine - Heroin - Meth - Other - IV - Inhalants - Other | - X Never - Past- year started/year quit - Current   - Quantity - # of years |
| Diet (describe) | All may be used |
| Exercise (describe) | An active toddler |
| List any other important social history or information important to this case | Dad lives out of state and is not yet aware Jimmy had this injury. Grandma is on her way to be with mom and Jimmy in the pediatric emergency department. |
| Family history |  |
| Mother, Father, Siblings, Grandparents, and other significant findings. | Not applicable to this exercise |
|  |  |
| Physical Exam-  3 cm gash near eyebrow | |
| PHYSICAL EXAM FINDINGS |  |
| 1. Written in layman’s terms | The toddler is not present during this exercise |
| 1. General appearance- affect, appearance, position of patient at opening (i.e. sitting, laying down, holding abdomen etc.) | Well |
| 1. Vital signs | 120 BPM, 106/74 BP, 22 RR, 100% O2 Sat |
| 1. Specific findings and affect | 3cm gaping laceration above Left eyebrow |
| 1. Response to certain physical movements | Cried as examiner approaches the child and even more so when the laceration was examined. |
|  |  |
| DIAGNOSIS AND DIFFERENTIAL |  |
| Diagnosis with support from positive and negative history and PE findings | 3 cm laceration near eyebrow through the dermis, mildly gaping |
| Differential with support from positive and negative history and PE findings | No signs of clinically important traumatic brain injury, c-spine trauma or other associated head, neck or facial injuries. |
|  |  |
| MANAGEMENT OR DIAGNOSTIC PLAN | LET placed on wound  Anxiolysis considerations such as Child Life and or Intranasal Midazolam  Laceration repair |
|  |  |
| PROFESSIONALISM ISSUES OR CHALLENGES: | Resident does not come off as confident or is overconfident  Resident uses too much medical jargon  Resident does not check for understanding well  Resident does not explain the steps of the procedure well  Resident is disorganized  Resident is not prepared to answer questions related to which wounds are best handled by a plastic surgeon |
