# Supplementary material for: Formative Assessments Promote Procedural Learning and Engagement for Senior Pediatric Residents on Rotation in the Pediatric Emergency Department
Source: MedEdPORTAL. 2022 Jul 12;18:11265. doi: 10.15766/mep_2374-8265.11265 (PMC9273678; doi:10.15766/mep_2374-8265.11265)
Supplement: Supplementary file 1 — LP OSCE.docxLAC OSCE.docxPSIM Equipment List.docxPre-Post Questionnaire.docxFormative Feedback Report.docx [file mep_2374-8265.11265-s001.zip › C. PSIM Equipment List.docx]

**Appendix C – Equipment Inventory for Procedural Simulations (PSIMs)**

1. **General Materials Needed for both PSIMs**

- Gloves
- Purell
- iPhone focused perpendicularly to resident’s hands on PSIM equipment


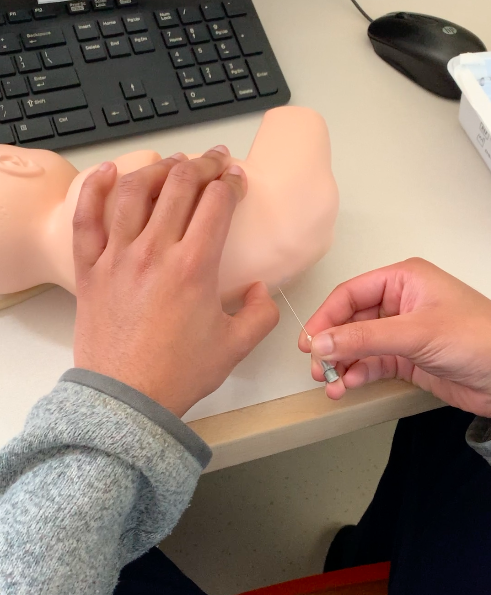

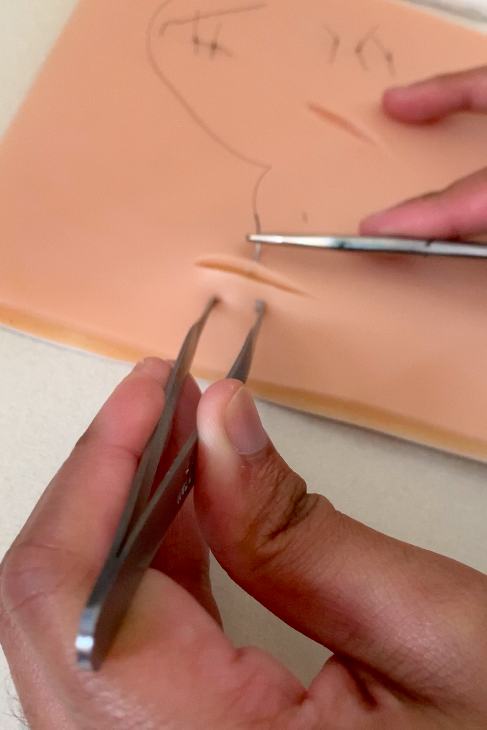


- Place all needed equipment below a drape prior to beginning of PSIM

1. **Laceration Station:**

- Instructions to state to the resident:
  - You have already consented the caregiver of a toddler who needs a forehead laceration repair.
  - On the sheet of paper (or verbalize), please place all orders you may want nursing to perform for you. I will be your RN.
  - Once you are ready, please lift the drape and perform all the necessary steps you would take to complete the procedure as if this were a real patient– from positioning to wound dressing.
    - You may verbalize as much as you typically verbalize with a real patient.
  - Place 2-3 simple interrupted sutures
- Equipment -
  - Laceration training pad
  - Lidocaine 1% w/ Epi; Lidocaine 1% w/o epi, Lidocaine 2% w and w/o epi
  - Blunt needle
  - 25g needle
  - 3 or 5cc syringe
  - 4.0, 5.0 and 6.0 sutures of varying types
  - Laceration kit
  - Basin
  - Sterile Water / Saline
  - Irrigation syringe
  - Irrigation syringe shield
  - Bacitracin
  - Gauze
  - Tape

**LP Station:**

- Instructions:
  - You have already consented the caregiver of a neonate who needs a lumbar puncture.
  - On the sheet of paper (or verbalize), please place all orders you may want nursing to perform for you. I will be your RN.
  - Once you are ready, please lift the drape and perform all the necessary steps you would take to complete the procedure as if this were a real patient– from positioning to wound dressing. I am your assistant as well and can help with positioning you may need.
    - You may verbalize as much as you typically verbalize with a real patient.
- Equipment –
  - Infant LP Simulator
  - Water
  - Lidocaine 1%
  - Blunt needle
  - 25g needle
  - 3 or 5cc syringe
  - 22g, 1.5in LP needle
  - LP kit
  - Betadine
  - Specimen bag
  - Mask
  - Sterile Gloves
  - Gauze
  - Band-Aid
