# Supplementary material for: Formative Assessments Promote Procedural Learning and Engagement for Senior Pediatric Residents on Rotation in the Pediatric Emergency Department
Source: MedEdPORTAL. 2022 Jul 12;18:11265. doi: 10.15766/mep_2374-8265.11265 (PMC9273678; doi:10.15766/mep_2374-8265.11265)
Supplement: Supplementary file 1 — LP OSCE.docxLAC OSCE.docxPSIM Equipment List.docxPre-Post Questionnaire.docxFormative Feedback Report.docx [file mep_2374-8265.11265-s001.zip › D. Pre-Post Questionnaire.docx]

Appendix D: Resident PRE/POST Questionnaire

As a reminder, the participation in the educational initiative is mandatory for all PGY2 and 3s, but the video recording of your performance and the filling out of these very brief questionnaires are most appreciated but completely voluntary. The study has been deemed exempt by the IRB and has been approved by the Residency Oversight Committee.

Unique Identifier: Please enter your easy to remember unique identifier: 6 digits of your birthday + first and last name initials Example: 082382mg

________________________________________________________________

For a toddler with a forehead laceration, please state your level of comfort independently performing the following components of the procedure:

|  | Very Low (1) | Low (2) | Neutral (3) | High (4) | Very High (5) |
| --- | --- | --- | --- | --- | --- |
| 1> Consenting the patient/caregiver (1) |  |  |  |  |  |
| 2> Formulating an analgesia plan (2) |  |  |  |  |  |
| 3> Formulating an anxiolysis plan (3) |  |  |  |  |  |
| 4> Organizing the procedural care team (i.e. giving guidance to your assistant / holder). (4) |  |  |  |  |  |
| 5> Determining the proper positioning of the patient (5) |  |  |  |  |  |
| 6> With the psychometric maneuvers to complete a laceration repair (6) |  |  |  |  |  |
| 7> Anticipating potential complications in common procedures (7) |  |  |  |  |  |
| 8> Completing a laceration repair completely independently (8) |  |  |  |  |  |

For a neonate needing a lumbar puncture, please state your level of comfort independently performing the following components of the procedure:

|  | Very Low (1) | Low (2) | Neutral (3) | High (4) | Very High (5) |
| --- | --- | --- | --- | --- | --- |
| 1> Consenting the patient/caregiver (1) |  |  |  |  |  |
| 2> Formulating an analgesia plan (2) |  |  |  |  |  |
| 3> Formulating an anxiolysis plan (3) |  |  |  |  |  |
| 4> Organizing the procedural care team (i.e. giving guidance to your assistant / holder). (4) |  |  |  |  |  |
| 5> Determining the proper positioning of the patient (5) |  |  |  |  |  |
| 6> With the psychometric maneuvers to complete a lumbar puncture (6) |  |  |  |  |  |
| 7> Anticipating potential complications in common procedures (7) |  |  |  |  |  |
| 8> Completing a lumbar puncture completely independently (8) |  |  |  |  |  |

At this moment, please select where you believe you fall on the following procedural trust scale. It measures the amount of trust you, the resident, should have when completing a forehead laceration on a toddler:

- Level 1: Resident trusted to observe the laceration repair (1)
- Level 2: Resident trusted to practice the laceration repair only under proactive, full supervision as a co-activity with the supervisor (2)
- Level 3: Resident trusted to practice the laceration repair only under proactive, full supervision with the supervisor in the room and ready to step in as needed (3)
- Level 4: Resident trusted to practice the laceration repair only under reactive, on-demand supervision with supervisor immediately available and ALL findings double checked (4)
- Level 5: Resident trusted to practice the laceration repair only under reactive, on-demand supervision with supervisor immediately available and KEY findings double checked (5)
- Level 6: Resident trusted to practice the laceration repair only under reactive, on-demand supervision with supervisor distantly available (eg, by phone), findings reviewed (6)
- Level 7: Resident trusted to practice the laceration repair unsupervised (7)
- Level 8: Resident trusted to supervise others in practice of the laceration repair (where supervision means: ability to assess patient and learner needs ensuring safe, effective care and further trainee development by tailoring supervision level) (8)

At this moment, please select where you believe you fall on the following procedural trust scale. It measures the amount of trust you, the resident, should have when completing a lumbar puncture on a neonate:

- Level 1: Resident trusted to observe the LP (1)
- Level 2: Resident trusted to practice the LP only under proactive, full supervision as a co-activity with the supervisor (2)
- Level 3: Resident trusted to practice the LP only under proactive, full supervision with the supervisor in the room and ready to step in as needed (3)
- Level 4: Resident trusted to practice the LP only under reactive, on-demand supervision with supervisor immediately available and ALL findings double checked (4)
- Level 5: Resident trusted to practice the LP only under reactive, on-demand supervision with supervisor immediately available and KEY findings double checked (5)
- Level 6: Resident trusted to practice the LP only under reactive, on-demand supervision with supervisor distantly available (eg, by phone), findings reviewed (6)
- Level 7: Resident trusted to practice the LP unsupervised (7)
- Level 8: Resident trusted to supervise others in practice of the LP (where supervision means: ability to assess patient and learner needs ensuring safe, effective care and further trainee development by tailoring supervision level) (8)

Please respond to the following prompts on a scale of Strongly Disagree to Strongly Agree: I am confident:

|  | Strongly Disagree (1) | Disagree (2) | Neutral (3) | Agree (4) | Strongly Agree (5) |
| --- | --- | --- | --- | --- | --- |
| 1> Self-reflecting on the level of entrustment I should have for common pediatric procedures (1) |  |  |  |  |  |
| 2> Discussing the level of entrustment I desire with my preceptor (2) |  |  |  |  |  |

Finally, please respond to the following prompts on a scale of Strongly Disagree to Strongly Agree: This learning intervention (OSCE + SIM + FFR):

|  | Strongly Disagree (1) | Disagree (2) | Neutral (3) | Agree (4) | Strongly Agree (5) |
| --- | --- | --- | --- | --- | --- |
| 1> Was a valuable use of my time (1) |  |  |  |  |  |
| 2> Was needed in my pediatric residency training (4) |  |  |  |  |  |
| 3> Improved my understanding of common pediatric procedures (2) |  |  |  |  |  |
| 4> Improved my approach to common pediatric procedures (5) |  |  |  |  |  |
| 5> Improved my independence in performing common pediatric procedures (6) |  |  |  |  |  |

As a result of this learning intervention I plan to make the following changes in my procedural practice:

________________________________________________________________

________________________________________________________________

The learning intervention could be more successful if:

________________________________________________________________

________________________________________________________________
