# Supplementary material for: Formative Assessments Promote Procedural Learning and Engagement for Senior Pediatric Residents on Rotation in the Pediatric Emergency Department
Source: MedEdPORTAL. 2022 Jul 12;18:11265. doi: 10.15766/mep_2374-8265.11265 (PMC9273678; doi:10.15766/mep_2374-8265.11265)
Supplement: Supplementary file 1 — LP OSCE.docxLAC OSCE.docxPSIM Equipment List.docxPre-Post Questionnaire.docxFormative Feedback Report.docx [file mep_2374-8265.11265-s001.zip › E. Formative Feedback Report.docx]

Appendix E: Formative Feedback Report for Resident__________

Dear Resident,

You recently completed a procedure-based OSCE and SIM. Thank you for your time and efforts to complete this educational initiative. Upon review of the videos generated of your performance, please accept this report as your formative feedback. This report is based on previously published procedural assessment scales.

We conclude this feedback report with two components:

1. What we perceive the level of entrustment you will need for similar procedures going forward.

2. General commentary not explicitly addressed in the assessment checklists

Resident Name

________________________________________________________________

| Procedural Consent – Lac repair (Kempner, et al)^1^ | Done (1) | Not Done (2) |
| --- | --- | --- |
| Stated that wounds are closed to minimize scaring (1) |  |  |
| Stated that wound are closed to prevent wound infection (2) |  |  |
| Explained the patient will be positioned in a position of comfort that best allows the procedure to be completed safely (3) |  |  |
| Explained sterile/aseptic techniques will be used (to clean the skin) (4) |  |  |
| Explained the patient will be given an anxiolytic – (e.g., IN Midazolam, Child Life Specialist) (5) |  |  |
| Explained the patient will be given something for pain control (e.g., LET and or lidocaine) (6) |  |  |
| Explained that the wound will be irrigated to minimize wound infection and remove any small foreign bodies (7) |  |  |
| Explained that a needle will be used to place the sutures to bring the skin edges together (8) |  |  |
| Explained that topical antibiotics and or a bulky dressing will be applied (9) |  |  |
| Explained that the sutures may dissolve or be removed by the pediatrician in 5-10 days (10) |  |  |
| Stated risk of possible introduction of infection (11) |  |  |
| Stated risk of minimal bleeding (12) |  |  |
| Stated risk of pain (13) |  |  |
| Explained alternative that the skin will eventually form a scar on its own (14) |  |  |
| Stated that no intervention is an option (15) |  |  |
| Explained the risk that no intervention has the disadvantages of risk of uncontrolled infection or poor cosmetics (16) |  |  |
| Explained the consent form point by point (17) |  |  |
| Checked for understanding (18) |  |  |
| Asked for signature on the consent form (19) |  |  |

| Lac Repair Competency Scale (Seo et al)^2^ | Done (1) | Not Done (2) |
| --- | --- | --- |
| Prepares for laceration: Selects appropriate type and size of needle, obtains laceration tray, kidney basin, and 60 mL syringe with cap for washout (1) |  |  |
| Positioning: Asks to position patient and laceration in a position such that the laceration is easy to access and is well lit (2) |  |  |
| Anesthetizes: Dons gloves, infiltrates laceration completely with 1% buffered lidocaine through the wound or tests for anesthesia if LET only (3) |  |  |
| Cleanses: Washes or pretends to wash laceration with 60 mL of water per 1 cm of laceration (4) |  |  |
| Maintains sterility: Drapes around laceration to create a sterile field and sterility is maintained throughout procedure (5) |  |  |
| Needle holder in the palm of the hand with index finger extended (6) |  |  |
| Forceps held in non-dominant hand (7) |  |  |
| Needle loaded onto needle holder between proximal and middle third of needle (8) |  |  |
| Needled enters skin perpendicular to the skin (9) |  |  |
| Exit at opposing end of laceration approximately equidistant from entrance bite (10) |  |  |
| Leaves a short (3 cm) tail of suture for tying (11) |  |  |
| Checks for appropriate wound approximation (12) |  |  |
| When tying, hemostat placed in the center of the laceration for tying (13) |  |  |
| Ties first knot with two loops (14) |  |  |
| Lays suture knot lateral to the laceration (15) |  |  |
| Completes suture tie by alternating hand positions, approximately 4 knots per suture (16) |  |  |
| Makes corrections If suture is incorrectly placed, i.e., wound does not approximate well, suture breaks, the suture is removed and resident starts over (17) |  |  |
| Discards sharps: Needle(s) safely/correctly handled and discarded (18) |  |  |

After review of your videos, we believe you fall on the following procedural trust scale. It measures the amount of trust you, the resident, should have when completing a forehead laceration on a toddler (Schumacher et al)^3^:

- Level 1: Resident trusted to observe the laceration repair (1)
- Level 2: Resident trusted to practice the laceration repair only under proactive, full supervision as a co-activity with the supervisor (2)
- Level 3: Resident trusted to practice the laceration repair only under proactive, full supervision with the supervisor in the room and ready to step in as needed (3)
- Level 4: Resident trusted to practice the laceration repair only under reactive, on-demand supervision with supervisor immediately available and ALL findings double checked (4)
- Level 5: Resident trusted to practice the laceration repair only under reactive, on-demand supervision with supervisor immediately available and KEY findings double checked (5)
- Level 6: Resident trusted to practice the laceration repair only under reactive, on-demand supervision with supervisor distantly available (eg, by phone), findings reviewed (6)
- Level 7: Resident trusted to practice the laceration repair unsupervised (7)
- Level 8: Resident trusted to supervise others in practice of the laceration repair (where supervision means: ability to assess patient and learner needs ensuring safe, effective care and further trainee development by tailoring supervision level) (8)

| Procedural Consent – LP (Kempner et al)^1^ | Done (1) | Not Done (2) |
| --- | --- | --- |
| Explained that meningitis is an infection of the covering membrane of the brain (1) |  |  |
| Explained that symptoms of fever may suggest meningitis in a newborn (2) |  |  |
| Explained the outcome(s) if untreated (e.g., brain damage including paralysis, mental disability, deafness, blindness or death) (3) |  |  |
| Stated the benefits of the LP as appropriate to diagnosis for meningitis (4) |  |  |
| Stated the ability to determine appropriate antibiotic treatment (5) |  |  |
| Explained that the patient will be positioned lying on his/her side (or seated at edge of bed) (6) |  |  |
| Explained that sterile/aseptic techniques will be used (to clean the skin) (7) |  |  |
| Explained that something for pain control will be used (local numbing medicine) (8) |  |  |
| Explained that something for anxiolysis will be given (e.g., sweeties) (9) |  |  |
| Explained that a needle will be placed in the spine (10) |  |  |
| Explained that spinal fluid will be removed (11) |  |  |
| Explained that the needle will be removed (12) |  |  |
| Stated the risk of possible introduction of infection to spinal fluid resulting in meningitis (13) |  |  |
| Explained that the risk of introducing infection in very rare (14) |  |  |
| Stated the risk of possible spinal cord injury (15) |  |  |
| Explained that the risk of spinal cord injury is very rare as needle is inserted below the cord (16) |  |  |
| Explained the alternative of administering antibiotics without LP done (17) |  |  |
| Explained the disadvantage of no LP as uncertainty of what is being treated or requires long hospital stay (18) |  |  |
| Stated that no intervention is an option (19) |  |  |
| Explained the disadvantage that no LP puts baby at risk of uncontrolled infection, seizures, death (20) |  |  |
| Explained the consent form to me point by point (21) |  |  |
| Checked for understanding (22) |  |  |
| Asked for my signature on the consent form (23) |  |  |

| Competency Scale – LP (Auerbach et al)^4^ | Done (1) | Not Done (2) |
| --- | --- | --- |
| Plans insertion site: Palpates iliac crest and follows to the midline interspace of L4/5 or L5-S1 on spine before beginning procedure (1) |  |  |
| Dons gloves, opens tray, selects appropriate needle (22G 1.5”) and opens/prepares tubes (2) |  |  |
| Discusses analgesia and anxiolysis (oral sucrose, topicals, lidocaine infiltration, sedation) (3) |  |  |
| Cleanses: (A) Betadine applied in 3 widening concentric circles (dry for 1- minute) OR (B) Chlorhexadine scrubbed for 30 seconds (4) |  |  |
| Maintains sterility: Drapes placed under and on top of model to create a sterile field and sterility is maintained throughout procedure (5) |  |  |
| Instructs holder: Asks to position model in lateral decubitus or sitting position in the fetal position without obstructing the airway (6) |  |  |
| Inserts needle at proper interspace L4/5 or L5/S1 (7) |  |  |
| Inserts in the midline of back at center of imaginary line drawn from iliac crest - iliac crest (8) |  |  |
| Inserted perpendicular into skin- relative to imaginary line from crest-crest (9) |  |  |
| Advances needle Toward umbilicus (~15 degrees cephalad) (10) |  |  |
| Advances one motion, avoids side to side or coarse movements (11) |  |  |
| Advances slowly with stylet intermittently removed to check for fluid OR stylet removed and kept out once needle is through the skin (12) |  |  |
| Makes corrections If no fluid obtained (e.g., rotates needle 90 degrees or slowly withdraws without exiting skin and redirects needle – avoids coarse movements) (13) |  |  |
| Acquires fluid: Each tube filled to approximately 0.5-1 ml (14) |  |  |
| Removes needle: Stylet replaced before needle is removed from skin and applies pressure with gauze (15) |  |  |
| Discards sharps: Needle safely/correctly handled and discarded (16) |  |  |

After review of your videos, we believe you believe you fall on the following procedural trust scale. It measures the amount of trust you, the resident, should have when completing a lumbar puncture on a neonate (Schumacher et al)^3^:

- Level 1: Resident trusted to observe the LP (1)
- Level 2: Resident trusted to practice the LP only under proactive, full supervision as a co-activity with the supervisor (2)
- Level 3: Resident trusted to practice the LP only under proactive, full supervision with the supervisor in the room and ready to step in as needed (3)
- Level 4: Resident trusted to practice the LP only under reactive, on-demand supervision with supervisor immediately available and ALL findings double checked (4)
- Level 5: Resident trusted to practice the LP only under reactive, on-demand supervision with supervisor immediately available and KEY findings double checked (5)
- Level 6: Resident trusted to practice the LP only under reactive, on-demand supervision with supervisor distantly available (eg, by phone), findings reviewed (6)
- Level 7: Resident trusted to practice the LP unsupervised (7)
- Level 8: Resident trusted to supervise others in practice of the LP (where supervision means: ability to assess patient and learner needs ensuring safe, effective care and further trainee development by tailoring supervision level) (8)

We offer the following additional feedback on your performance, please accept it with our full understanding of the limits of SIM and how being video recorded may influence your actual performance on real patients:

________________________________________________________________

________________________________________________________________

________________________________________________________________

________________________________________________________________

________________________________________________________________

****Please note, competency checklists and EPA assessment scales have been adapted from the originally published tools to best fit the Formative Feedback Report.***

Citations:

1. Kempner S, Morgan H, Stern D, et al. Providing Informed Consent: A Standardized Case. *MedEdPORTAL Publ*. 2016;12(12). doi:10.15766/mep_2374-8265.10427

2. Seo S, Thomas A, Uspal N. A Global Rating Scale and Checklist Instrument for Pediatric Laceration Repair. *MedEdPORTAL*. 2019;15(15). doi:10.15766/mep_2374-8265.10806

3. Schumacher DJ, West DC, Schwartz A, et al. Longitudinal Assessment of Resident Performance Using Entrustable Professional Activities. *JAMA Netw open*. 2020;3(1):e1919316. doi:10.1001/jamanetworkopen.2019.19316

4. Auerbach M, Chang T, Fein D, et al. A Comprehensive Infant Lumber Puncture Novice Procedural Skills Training Package: An INSPIRE Simulation-Based Procedural Skills Training Package. *MedEdPORTAL Publ*. 2014;(10). doi:10.15766/mep_2374-8265.9724
