# Supplementary material for: Formative Assessments Promote Procedural Learning and Engagement for Senior Pediatric Residents on Rotation in the Pediatric Emergency Department
Source: MedEdPORTAL. 2022 Jul 12;18:11265. doi: 10.15766/mep_2374-8265.11265 (PMC9273678; doi:10.15766/mep_2374-8265.11265)
Supplement: Supplementary file 1 — LP OSCE.docxLAC OSCE.docxPSIM Equipment List.docxPre-Post Questionnaire.docxFormative Feedback Report.docx [file mep_2374-8265.11265-s001.zip › Educational_Summary_Report_11265.pdf]

MedEdPORTAL® The AAMC Journal of Teaching and Learning Resources

Original Publication Open Access

Formative Assessments Promote Procedural Learning and Engagement for Senior Pediatric Residents on Rotation in the Pediatric Emergency Department

Michael P. Goldman, MD*, Alexis V. Rudd, MD, Sophie C. Baum, Madeline Nagler, Doria L. Weiss, Isabel T. Gross, MD, PhD, MPH, Marc A. Auerbach, MD, MSc

*Corresponding author: michael.goldman@yale.edu

Abstract

Introduction: Procedural training is a universal concern amongst pediatric residents and their teachers. We developed and implemented formative assessments to generate direct and indirect procedural feedback. We analyzed changes in residents' perceived procedural knowledge, skills, confidence, and entrustment. **Methods:** Senior pediatric residents rotating in the pediatric emergency department participated in video-recorded formative assessments of informed consent OSCEs and simulated toddler forehead laceration repair and infant lumbar puncture. Residents reflected on their perceived procedural knowledge, skills, confidence, and entrustment through Likert and entrustment scales. Secondary outcomes of formative assessment completion rates and proportions of procedures performed by pediatric residents tracked feasibility and potential clinical impact, respectively. **Results:** Including the pilot period, 89% of residents (31 out of 35) received direct and indirect procedural feedback. Perceived composite competency and entrustment improved for laceration repair (competency: from 3.1 to 3.9, $p < .001$; entrustment: from 4.0 to 5.1, $p < .001$) and lumbar puncture (competency: from 3.5 to 4.0, $p < .001$; entrustment: from 4.6 to 5.6, $p = .001$). We observed an increase in the proportion of clinical laceration repairs (11% [97 out of 885] vs. 23% [218 out of 946], $p < .001$) and lumbar punctures (23% [12 out of 54] vs. 41% [21 out of 52], $p = .05$) performed by pediatric residents. **Discussion:** Integrating feasible procedural formative assessments into the pediatric emergency department rotation had a positive impact on senior pediatric residents' perceptions of their procedural knowledge, skills, confidence, and entrustment and was associated with increased procedural engagement.

Keywords
Clinical Skills Assessment/OSCEs, Clinical/Procedural Skills Training, Competency-Based Medica Education (Competencies, Milestones, EPAs), Pediatric Emergency Medicine, Pediatrics, Simulation

Educational Objectives

After participation in this series of formative assessments, senior pediatric residents will be able to:

1. Develop their perceived knowledge, skills, and confidence in performing two common pediatric procedures as measured by pre- and postparticipation questionnaires.
2. Demonstrate their ability to obtain informed consent for two common pediatric procedures with standardized caregivers through participation in OSCE formative assessments.
3. Practice the psychomotor maneuvers for two common pediatric procedures on simulation equipment by completing workplace-based formative assessments.
4. Develop their ability to reflect on procedural entrustment as measured by pre- and postparticipation questionnaires.

Citation:
Goldman MP, Rudd AV, Baum SC, et al. Formative assessments promote procedural learning and engagement for senior pediatric residents on rotation in the pediatric emergency department. *MedEdPORTAL*. 2022;18:11265.
https://doi.org/10.15766/mep_2374-8265.11265

Introduction

For a resident to successfully graduate from pediatric residency, the Accreditation Council for Graduate Medical Education (ACGME) states that program directors must deem the resident competent in the procedures typically used by a general pediatrician, including simple laceration repair (LAC) and infant

Copyright © 2022 Goldman et al. This is an open-access publication distributed under the terms of the Creative Commons Attribution license.

lumbar puncture (LP).[1] However, there is considerable variation in quantifying, assessing, and documenting competency.[2,3]

For a myriad of reasons, there is wide heterogeneity in resident exposure to and comfort levels with performing procedures.[4-6] Furthermore, supervision can be irregular, depending on clinical unit and supervisor's experience.[4] These factors impact opportunity to practice obtaining informed consent, a core element of the procedural entrustable professional activity.[7,8] Highlighting this problem, one study shared that 27% of graduating pediatric residents have never performed an LAC and 32% have never performed an LP.[2] This results in poor procedural outcomes for pediatric trainees, exemplified by an LP study where, despite a robust training modality, graduating pediatric residents had a 54% first-pass success rate.[4]

When residents do perform procedures, supervisor feedback is often informal and may lack the detail required to improve future performance.[9] To our knowledge, few, if any, pediatric residency programs employ formative assessments (FAs) to assess procedural competency and standardize feedback. Thus, an urgent need remains for pediatric residency programs to incorporate both structured opportunities for practice and objective feedback.

Structured, as opposed to ad hoc, FAs are gaining momentum in medical training as an approach to cultivate deep learning through the use of low-stakes, frequent, learner-focused feedback.[10] FAs differ from summative assessments by emphasizing assessment for learning over assessment of learning.[10] Essentially, the FA offers the student and teacher both specific and generalizable gaps to guide learning, a pedagogical approach purposefully incorporated into our program to meet each learner's individual needs.[11]

The objective structured clinical exam (OSCE) is a platform that provides both formative and summative assessment of trainees.[12,13] Simulation offers the learner situations for education and practice and can augment procedural knowledge development and competency and combat clinical performance anxiety.[14-16]

Trainee feedback from preceptors can occur through several formats, either directly during the learning experience (i.e., coaching) or indirectly through unidirectional feedback reports or bidirectional debriefing sessions. Feedback can be facilitated by tools such as global rating scales, competency checklists, and, more recently, entrustment scales.[17-20] Each assessment tool has its strengths and weaknesses; some investigators have commented that a competency checklist appears more appropriate for technical skills whereas global rating scales or entrustment scales may be more appropriate for communication skills, teamwork, and the holistic skill of doctoring.[12,19,20]

To address the current landscape of pediatric procedural training at our institution, we developed a series of FAs to generate standardized procedural feedback. We aimed to improve senior pediatric residents' perceived procedural knowledge, skills, and confidence while exploring the impact on entrustment. We designed our FA program balancing realism with feasibility to promote implementation and sustainability. Furthermore, we incorporated several procedure-related resources from *MedEdPORTAL,* utilized FAs as the primary teaching modality, and incorporated the emerging entrustable professional activities assessment construct.[8,16,18,21,22] Finally, we explored whether our FAs were associated with increased pediatric resident procedural experiences in the clinical setting.

Through our outcomes reported in conjunction with reflections from resident participants and facilitators, we present a well-received, low-cost, and feasible procedural FA program that can be readily implemented and sustained. The target audience of the procedural FAs was senior (PGY 2-4) pediatric and med/peds residents as, in contrast to interns, the expectations for procedural entrustment of senior residents were less predictable.[23]

Methods

Context

Senior resident participation in the FAs was required by the pediatric residency and pediatric emergency department (PED) educational leadership because our local ACGME review echoed the national procedural training concern. To facilitate a standardized feedback process and to promote feasibility, we recorded all OSCE and procedural simulation (PSIM) FAs to allow the lead facilitator (Michael P. Goldman) to perform indirect observation and generate feedback reports. Our work was approved by our institution's Internal Review Board and our residency program's Research Oversight Committee.

At our institution, most pediatric residents rotate through the PED annually for 4 consecutive weeks. Each year, our PED sees approximately 36,000 patients, including an estimated 1,100 LACs and 80 LPs. A typical shift is staffed by two pediatric emergency medicine (PEM) attendings, one PEM fellow, one emergency medicine resident, one pediatrics intern, and one pediatric senior resident. Most months average three senior pediatric residents on rotation. Prior to participation in the

Copyright © 2022 Goldman et al. This is an open-access publication distributed under the terms of the Creative Commons Attribution license.

MedEdPORTAL® The AAMC Journal of Teaching and Learning Resources

FAs, the only prerequisite learning requirement was that all participants rotated through the PED intern year.

To control costs, we trained volunteers to act as standardized caregivers (SCs) for the informed consent OSCEs. The three SCs were colleagues' family members interested in health science school matriculation (Sophie C. Baum, Madeline Nagler, and Doria L. Weiss). Incentives to participate as SCs included authorship. Training of SCs included a session and an observed pilot OSCE. The hour-long training session was facilitated by a colleague in our simulation center who had both standardized patient and professional acting experience. Training emphasized avoiding formal scripting in favor of acting as naturally as possible in response to the residents' words and actions, as if the SCs were bringing their own relative to the PED. We reviewed each procedure and discussed commonly encountered caregiver questions and concerns. The SCs' training session concluded with opportunities to practice among themselves using the OSCEs (Appendices A and B). These included instructions to repeat verbatim to initiate the exercise and challenge questions to facilitate a realistic informed consent process. We did not train the SCs to grade resident performance or make a formal determination on granting informed consent. Rather, we asked them to allow the resident to initiate the procedure. The three SCs were women in their mid-20s without any formal medical background.

PSIM facilitators (Michael P. Goldman, Isabel T. Gross, and Marc A. Auerbach) met to promote standardization. We agreed upon instructions such as the angle at which to film the resident's hands, when and how to offer direct feedback, and what to stock in the equipment cart (Appendix C).[24] After extensive piloting, we decided that immediate feedback was appropriate and beneficial to the resident. We discouraged any coaching during recording but encouraged deliberate practice after. Additionally, piloting led to minor iterative changes to the pre-post questionnaire (Appendix D) and the instructions for the OSCEs and PSIMs. The three PSIM facilitators were all PEM faculty with extensive simulation and educational experience.

Learner Assessment

The FA program followed the procedure shown in the Figure. First, the resident completed the prequestionnaire prior to their scheduled informed consent OSCEs. OSCEs were completed from home via Zoom (Appendix D). Each OCSE took approximately 10 minutes per case. Next, facilitators proctored PSIMs during subsequent PED shifts. The PSIM took approximately 7.5 minutes to set up and complete for each procedure. Upon completion of the PSIM, the resident

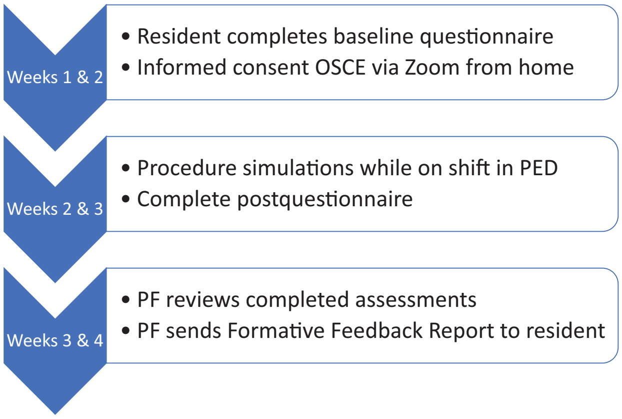

Figure. The formal assessment program. Abbreviations: PED, pediatric emergency department; PF, primary facilitator.

immediately filled out the postquestionnaire and then received feedback from their facilitator. During the third week of the PED rotation, the lead facilitator reviewed all four videos and completed a Formative Feedback Report (FFR). This report included previously published competency checklists[8,18,22] and entrustment scales[23] and allowed space for free-text commentary (Appendix E). The competency checklists were provided to the resident in the FFR to ground qualitative feedback from the primary facilitator who watched their performance on video. In-person PSIM facilitators could use those same checklists to ground verbal feedback, but those copies were not filled out or handed to the resident as a score or grade. If the competency checklist stem was double- or triple-barreled, facilitators would mark them "completed," with room to expand on the resident's performance via free text.

Outcomes and Analysis Plan

Pre- and postquestionnaires measured residents' perceived knowledge, skills, confidence, and entrustment in performing the two core procedures (Appendix D). Paired *t* tests compared the near-parametric primary outcomes of mean 5-point Likert-scale responses and 8-point entrustment scores.[23] The 5-point Likert scale ranged from very low (1) to very high (5) comfort in independently performing key components of the respective procedure. Each procedure generated a composite score averaging these components.

Additional outcomes reported include data gathered during piloting. These data explored residents' reactions to how the FAs impacted self-reflection on their procedural autonomy, solicited constructive feedback on the FA program, and invited insight into how the FAs influenced residents' procedural practice in the clinical setting. The data comparing near-parametric pre-post

Copyright © 2022 Goldman et al. This is an open-access publication distributed under the terms of the Creative Commons Attribution license.

5-point Likert-scale responses are reported via unpaired *t* tests, chi-square tests, descriptive statistics, and thematic analysis.

Feasibility was measured through report of the FA completion rate during the formal enrollment period and as the total percentage of senior residents who received FFRs, including during the pilot period.

Descriptive data from the FFRs highlighted the most commonly omitted steps from the OSCE and PSIM competency checklists, excluding double- or triple-barreled checklist probes. Three facilitators performed thematic analysis of the clinical changes residents planned to make as a result of the FAs and the residents' feedback on the FAs' experience and logistics.

Finally, the chi-square test compared the proportion of procedures completed by pediatric residents during the formal enrollment period with the similar months of the 2 years prior, given the well-documented impact of COVID-19 on PED census (i.e., August 2018-May 2019 and August 2019-May 2020 vs. August 2020-May 2021).[6]

Results

Of the 22 residents offering paired data for analysis of the primary outcomes, 86% reported liking procedures, and 32% were bound for a procedural-oriented career path. The median number of prior clinical LACs (with interquartile range [IQR] in parentheses) was 5.5 (IQR: 3.0-8.0) and, for LPs, was 5.0 (IQR: 3.0-6.0).

The primary outcomes outlining residents' perceptions of knowledge, skills, and confidence in obtaining informed consent and performing the two procedures are reported in Table 1. Statistically significant improvements were noted in each procedural component and in each procedure's composite scores. Furthermore, residents reported perceived improvements on the 8-point entrustment scale in both LAC (4.0 vs. 5.1, $p < .001$) and LP (4.6 vs. 5.6, $p = .001$).

With respect to feasibility, 86% of eligible residents (24 out of 28) completed all four FAs and received an FFR. Including piloting, 89% of residents (31 out of 35) received direct and indirect observation and an FFR.

Residents offered the following additional reflections on procedural entrustment. First, on a 5-point Likert scale (1 = *strongly disagree,* 5 = *strongly agree*), residents reported an increase in ability to self-reflect on their procedural autonomy (from 3.7 to 4.4, $p = .001$) and a trend towards an increase in confidence discussing the level of entrustment desired from their preceptors (from 3.9 to 4.3, $p = .06$).

Residents shared clinical changes they planned to make after participating in the FAs. Sixteen residents offered 19 responses, grouped into the following themes: psychomotor learning points ($n = 9$), entrustment considerations ($n = 5$), and developing a general approach to common procedures ($n = 5$). The psychomotor comments reflected specific techniques learned. One resident stated they would "use their wrist [more] to drive the needle through for LAC." The entrustment comments spoke to the procedural supervision discussions between residents and supervisors. A resident shared, "I'll be more proactive about seeking instruction from my supervisors via [simulation] before performing [clinical] procedures, especially if it has been a while." Finally, residents reported the FAs advanced their general approach to procedures. As one resident noted, the FAs "helped me think critically about everything I would need to do for a procedure from start to finish, including consent, materials,

Table 1. Paired Analysis of Mean Resident Responses

		Laceration Repair[b]			Lumbar Puncture[c]		
**Domain**	**Probe**[a]	**Pre**	**Post**	***p***	**Pre**	**Post**	***p***
Knowledge							
	Formulating an anxiolysis plan	3.2	3.9	<.001	3.5	3.9	.002
	Formulating an analgesia plan	3.2	4.0	<.001	3.6	4.1	.004
	Anticipating potential complications in common procedures	2.9	3.6	.004	3.4	3.7	.04
Skills							
	Consenting the patient/caregiver	4.1	4.5	.004	4.3	4.6	.02
	Organizing the procedural care team (i.e., giving guidance to your assistant/holder)	3.1	4.0	<.001	3.4	4.1	.002
	Determining the proper positioning of the patient	3.1	4.0	<.001	3.6	4.3	.001
	With the psychometric maneuvers to complete the procedure	2.8	3.9	<.001	3.3	4.0	.001
Confidence							
	Completing the procedure completely independently	2.4	3.5	<.001	2.9	3.5	<.001
Composite	Average	3.1	3.9	<.001	3.5	4.0	<.001

[a]Rated on a 5-point Likert scale (1 = *very low,* 5 = *very high*).
[b]For a toddler with a forehead laceration, state your level of comfort independently performing the following components of the procedure.
[c]For a neonate needing a lumbar puncture, state your level of comfort independently performing the following components of the procedure.

Copyright © 2022 Goldman et al. This is an open-access publication distributed under the terms of the Creative Commons Attribution license.

teammates and skills." Another added, "[the FAs taught] me a standardized approach for consent."

Reflecting on the FA pedagogical approach, respondents agreed or strongly agreed that the FAs improved their approach (74%, 31 out of 42), independence (81%, 33 out of 41), and understanding (76%, 31 out of 41) of common pediatric procedures. Additionally, respondents agreed or strongly agreed the FAs were a valuable use of their time (95%, 39 out of 41) and were needed in their residency training (88%, 36 out of 41).

Thirteen residents offered 13 comments on the FAs' experience and logistics. The three themes that emerged were general suggestions to improve procedural learning ($n = 5$), specific areas for improvement of the FA program ($n = 4$), and a general appreciation of the FAs ($n = 4$). An example of first theme was to "add short videos before or after to drive home best practices." An example of the second was to "consider doing this training during intern year, ideally in the beginning of the block." Finally, an example of the third theme was "it was wonderful receiving feedback about our skills in a safe, simulated environment!"

Review of 25 completed FFRs allowed for description of the most commonly omitted competencies for the informed consent and psychomotor skills of the included procedures (Table 2).[8,17,18,22]

Finally, we reviewed our dynamic procedure tracking tool (EPIC Systems Corp.) for the proportion of LACs and LPs performed in our PED by all pediatric residents (i.e., interns and senior residents). Comparing the enrollment period with the baseline procedural practice, we observed an increase in the proportion of LACs (11% [97 out of 885] vs. 23% [218 out of 946], $p < .001$) and LPs (23% [12 out of 54] vs. 41% [21 out of 52], $p = .05$) performed by pediatric residents.

Discussion

Integrating a low-cost, feasible procedural FA program into the PED rotation had a positive impact on residents' perceptions of procedural knowledge, skills, confidence, and entrustment. Furthermore, we observed an association with increased procedural engagement in the clinical setting. These FAs build upon best-practice training constructs such as the OSCE, just-in-time simulation, and standardized competency checklists to promote a rigorous training modality to a learning group with an identified gap. Additionally, as opposed to a more traditional pre-post learning exposure design, our approach highlights how FAs can generate meaningful individual and collective teaching points. Reflecting on the outcomes reported in conjunction with resident and facilitator feedback, we offer a feasible blueprint for colleagues to help address pediatric procedural learning.

First, the effectiveness of our approach is supported by the primary outcomes reported. While these data are low on the Kirkpatrick hierarchy[25] of medical education scholarship, they incorporate a novel pedagogical approach of using FAs to drive learning and pushed residents to self-reflect on procedural entrustment.[10,11,23,26] Although we report increased clinical procedural engagement, these data require more granularity to claim more than an association, as we do not know if the residents who participated in the FA program were the same residents who became more engaged in clinical procedures.

Second, at every planning juncture, we thought deeply about the balance between validity, realism, and feasibility. Paramount to our program was ensuring buy-in from busy residents. Thus, while acknowledging the deviation from reality, residents performed the informed consent OSCEs from home and the

Table 2. Competencies of Procedural Informed Consent and Psychomotor Skills Omitted by More Than 50% of Participants ($N = 25$)

Omitted Competencies	% Missed
During the LAC informed consent OSCE, residents did not state that:	
Wounds are closed to prevent wound infection	52
Patient will be positioned in a position of comfort that best allows the procedure to be completed safely	64
No intervention was an option	68
The risk that no intervention has the disadvantages of risk of uncontrolled infection or poor cosmetics	60
During the LAC PSIM, residents did not:	
Place needle holder in the palm of the hand with index finger extended	60
Lay suture knot lateral to the laceration	72
During the LP informed consent OSCE, residents did not state that:	
The outcome(s) [meningitis] if untreated (e.g., brain damage including paralysis, mental disability, deafness, blindness, or death)	56
No intervention was an option	80
The disadvantage that no LP puts baby at risk of uncontrolled infection, seizures, and death	72
During the LP PSIM, residents did not:	
Utilize the "early stylet out" or "Cincinnati technique"	76

Abbreviations: LAC, laceration repair; LP, lumbar puncture; PSIM, procedural simulation.

Copyright © 2022 Goldman et al. This is an open-access publication distributed under the terms of the Creative Commons Attribution license.

PSIMs in the clinical working environment.[27,28] Our data support that this logistical decision facilitated participation as 89% of residents completed the FAs and received an FFR. This feasibility figure is encouraging and is partially attributable to residency leadership buy-in. Yet we had aimed for 100% participation, which was impacted by two residents who were opposed to video recordings and two residents who could not complete all the tasks in the month. Based on resident and facilitator reflections, one could consider the option of audio recording the OSCEs and/or self-recording the PSIM. Either approach might assuage performance anxieties and improve resident participation. They might also improve logistics as only one facilitator would be needed to complete video reviews and FFRs.

Third, we offer additional reflections on potential impediments to implementation. The process for scheduling the residents with the SCs was not ideal, requiring several emails between the lead facilitator, the residents, and the SCs. Next, a secure, cloud-based storage server was needed as some videos proved too large for easy file-sharing. Finally, completion of the PSIMs required reminders between the residents and facilitators and was sometimes challenging during busy shifts. Thus, while we report some positive feasibility metrics related to acceptability, demand, and integration, we also share our deficits in practicality and have yet to demonstrate expansion.[29] Ideas to promote feasibility include a centralized scheduling dashboard, training more PSIM facilitators, or scheduling PSIMs right before a shift so as not to interfere with clinical responsibilities. As FAs aim to create low-stakes learning opportunities, any changes to promote participation by simplifying logistics are encouraged.

Next, there are important limitations to review, first, with respect to generalizability. These FAs were successfully implemented in a medium-size PED with the above-described staffing pattern. Clinical or other educational demands on residents at other institutions may impact participation in and perceptions of the learning.

With respect to validity, we used OSCEs and PSIMs to ensure all senior residents had a formative procedural learning experience. While similar approaches using clinical patients or trained actors may add realism, they were logistically impossible, as well as too expensive, and might have been too stressful for ideal learning.[10,11,27] Furthermore, our SCs were not professionals, which may have resulted in varied resident experiences. We embraced this reality, feeling that our approach to SC training produced a realistic enough experience to generate individualized feedback. We also did not collect potentially useful resident performance data from the SCs as time constraints limited more formal rater training. Moreover, because our primary outcomes data derive from nonvalidated questionnaires, we are vulnerable to biases.[30] It is also important to note that since a single facilitator completed the FFRs and our results do not include residents' reflection on the FFR, we cannot support or refute the FFR's utility in the FA program, though it did ensure each resident received standardized feedback.

Future directions for our work stem from resident feedback, facilitator reflections, and our need to adapt to new resident scheduling patterns. This year, the PED rotation is changing to two 2-week blocks. Adjustments may entail focusing on one resident class at a time or on one procedure at a time or dividing enrollment of the residents between their first and second rotations. As suggested by the residents themselves, we will add best-practice videos of informed consent and common pediatric procedures for reference but have yet to decide on when to integrate them into the FA program. Repeat FAs with select residents or synthesizing common FFR themes into a group learning activity could be another way to promote growth. Finally, we hope our FA approach is spread throughout the hospital in other departments where one could envision similar FA programs. Repetitive practice in diverse care settings should synergize to ensure development of an organized approach to informed consent and the basic tenets of any procedure. Thereafter, psychomotor practice can consider both the basic residency requirements[1] and a resident's career trajectory.

In summary, we offer the medical education community our approach and reflections addressing the national concern over procedural competency and autonomy upon graduating from pediatric training. We look forward to collaboration and are open to feedback on our FA program from the network of medical educators addressing this learning gap.

Appendices

A. LP OSCE.docx
B. LAC OSCE.docx
C. PSIM Equipment List.docx
D. Pre-Post Questionnaire.docx
E. Formative Feedback Report.docx

All appendices are peer reviewed as integral parts of the Original Publication.

Copyright © 2022 Goldman et al. This is an open-access publication distributed under the terms of the Creative Commons Attribution license.

Michael P. Goldman, MD: Assistant Professor, Departments of Pediatrics and Emergency Medicine, Section of Pediatric Emergency Medicine, Yale University School of Medicine; ORCID: https://orcid.org/0000-0003-1237-8820

Alexis V. Rudd, MD: Clinical Fellow, Department of Pediatrics, Division of Emergency Medicine, Nicklaus Children's Hospital

Sophie C. Baum: Third-Year Undergraduate Student, Barnard College of Columbia University

Madeline Nagler: Third-Year Undergraduate Student, Wesleyan University

Doria L. Weiss: Fourth-Year Undergraduate Student, University of Michigan

Isabel T. Gross, MD, PhD, MPH: Associate Professor, Departments of Pediatrics and Emergency Medicine, Section of Pediatric Emergency Medicine, Yale University School of Medicine

Marc A. Auerbach, MD, MSc: Professor, Departments of Pediatrics and Emergency Medicine, Section of Pediatric Emergency Medicine, Yale University School of Medicine

Acknowledgments
The authorship team would like to acknowledge Ms. Joy Grabow from the Yale University Medical School's Simulation Center who provided standardized caregiver training.

Disclosures
None to report.

Funding/Support
None to report.

Prior Presentations
Rudd AV, Auerbach MA, Goldman MP. An integrated procedural learning intervention in the pediatric emergency department. Poster presented virtually at: Pediatric Academic Societies Annual Meeting; May 2021.

Rudd AV, Auerbach MA, Goldman MP. An integrated procedural learning intervention in the pediatric emergency department. Poster presented virtually at: Yale School of Medicine Medical Education Day; May 2021.

Ethical Approval
The Yale Department of Pediatrics Residency Research Oversight Committee approved this project. The Yale University School of Medicine Institutional Review Board deemed further review of this project not necessary.

References

1. Accreditation Council for Graduate Medical Education. *ACGME Program Requirements for Graduate Medical Education in Pediatrics*. Accreditation Council for Graduate Medical Education; 2014.
2. Ben-Isaac E, Keefer M, Thompson M, Wang VJ. Assessing the utility of procedural training for pediatrics residents in general pediatric practice. *J Grad Med Educ*. 2013;5(1):88-92. https://doi.org/10.4300/jgme-d-11-00255.1
3. Bismilla Z, Dubrowski A, Amin HJ. Program directors' perceptions of importance of pediatric procedural skills and resident preparedness. *BMC Res Notes*. 2015;8:550. https://doi.org/10.1186/s13104-015-1499-8
4. Auerbach MA, White ML, Bhargava S, et al. Are graduating pediatric residents prepared to perform infant lumbar punctures? A multi-institutional descriptive study. *Pediatr Emerg Care*. 2018;34(2):116-120. https://doi.org/10.1097/PEC.0000000000000914
5. Grantcharov TP, Reznick RK. Teaching procedural skills. *BMJ*. 2008;336(7653):1129-1131. https://doi.org/10.1136/bmj.39517.686956.47
6. DeLaroche AM, Rodean J, Aronson PL, et al. Pediatric emergency department visits at US children's hospitals during the COVID-19 pandemic. *Pediatrics*. 2020;147(4):e2020039628. https://doi.org/10.1542/peds.2020-039628
7. ten Cate O, Carraccio C. Envisioning a true continuum of competency-based medical education, training, and practice. *Acad Med*. 2019;94(9):1283-1288. https://doi.org/10.1097/ACM.0000000000002687
8. Kempner S, Morgan H, Stern D, et al. Providing informed consent: a standardized case. *MedEdPORTAL*. 2016;12:10427. https://doi.org/10.15766/mep_2374-8265.10427
9. Williams RG, Klamen DA, McGaghie WC. Cognitive, social and environmental sources of bias in clinical performance ratings. *Teach Learn Med*. 2003;15(4):270-292. https://doi.org/10.1207/S15328015TLM1504_11
10. Rushton A. Formative assessment: a key to deep learning? *Med Teach*. 2005;27(6):509-513. https://doi.org/10.1080/01421590500129159
11. Epstein RM. Assessment in medical education. *N Engl J Med*. 2007;356(4):387-396. https://doi.org/10.1056/NEJMra054784
12. Newble D. Techniques for measuring clinical competence: objective structured clinical examinations. *Med Educ*. 2004;38(2):199-203. https://doi.org/10.1111/j.1365-2923.2004.01755.x
13. Iyer MS, Santen SA, Nypaver M, et al. Assessing the validity evidence of an objective structured assessment tool of technical skills for neonatal lumbar punctures. *Acad Emerg Med*. 2013;20(3):321-324. https://doi.org/10.1111/acem.12093
14. Craig SS, Auerbach M, Cheek JA, et al.; Pediatric Emergency Research Networks (PERN). Preferred learning modalities and practice for critical skills: a global survey of paediatric emergency medicine clinicians. *Emerg Med J*. 2018;36(5):273-280. https://doi.org/10.1136/emermed-2017-207384
15. Mills DM, Williams DC, Dobson JV. Simulation training as a mechanism for procedural and resuscitation education for

Copyright © 2022 Goldman et al. This is an open-access publication distributed under the terms of the Creative Commons Attribution license.

pediatric residents: a systematic review. *Hosp Pediatr*. 2013;3(2):167-176. https://doi.org/10.1542/hpeds.2012-0041

16. McMillan HJ, Writer H, Moreau KA, et al. Lumbar puncture simulation in pediatric residency training: improving procedural competence and decreasing anxiety. *BMC Med Educ*. 2016;16:198. https://doi.org/10.1186/s12909-016-0722-1
17. Gerard JM, Kessler DO, Braun C, Mehta R, Scalzo AJ, Auerbach M. Validation of global rating scale and checklist instruments for the infant lumbar puncture procedure. *Simul Healthc*. 2013;8(3):148-154. https://doi.org/10.1097/SIH.0b013e3182802d34
18. Seo S, Thomas A, Uspal NG. A global rating scale and checklist instrument for pediatric laceration repair. *MedEdPORTAL*. 2019;15:10806. https://doi.org/10.15766/mep_2374-8265.10806
19. Schumacher DJ, Frintner MP, Cull W. Graduating pediatric resident reports on procedural training and preparation. *Acad Pediatr*. 2018;18(1):73-78. https://doi.org/10.1016/j.acap.2017.08.001
20. ten Cate O, Carraccio C, Damodaran A, et al. Entrustment decision making: extending Miller's pyramid. *Acad Med*. 2021;96(2):199-204. https://doi.org/10.1097/ACM.0000000000003800
21. Mangold KA, Jeffers JM, Burns RA, et al. An objective structured clinical examination to improve formative assessment for senior pediatrics residents. *J Grad Med Educ*. 2015;7(3):470-474. https://doi.org/10.4300/JGME-D-14-00777.1
22. Auerbach M, Chang T, Fein D, et al. A comprehensive infant lumbar puncture novice procedural skills training package: an INSPIRE simulation-based procedural skills training package. *MedEdPORTAL*. 2014;10:9724. https://doi.org/10.15766/mep_2374-8265.9724
23. Schumacher DJ, West DC, Schwartz A, et al.; Association of Pediatric Program Directors Longitudinal Educational Assessment Research Network General Pediatrics Entrustable Professional Activities Study Group. Longitudinal assessment of resident performance using entrustable professional activities. *JAMA Netw Open*. 2020;3(1):e1919316. https://doi.org/10.1001/jamanetworkopen.2019.19316
24. Goldman MP, Palladino LE, Malik RN, et al. A workplace procedure training cart to augment pediatric resident procedural learning. *Pediatr Emerg Care*. 2022;38(2):e816-e820. https://doi.org/10.1097/pec.0000000000002397
25. Mills DM, Teufel RJ II. Tools for medical education scholarship: from curricular development to educational research. *Hosp Pediatr*. 2020;10(5):452-457. https://doi.org/10.1542/hpeds.2019-0293
26. Cutrer WB, Miller B, Pusic MV, et al. Fostering the development of master adaptive learners: a conceptual model to guide skill acquisition in medical education. *Acad Med*. 2017;92(1):70-75. https://doi.org/10.1097/ACM.0000000000001323
27. Pusic MV, Triola MM. Determining the optimal place and time for procedural education. *BMJ Qual Saf*. 2017;26(11):863-865. https://doi.org/10.1136/bmjqs-2017-007120
28. Sawyer T, White M, Zaveri P, et al. Learn, see, practice, prove, do, maintain: an evidence-based pedagogical framework for procedural skill training in medicine. *Acad Med*. 2015;90(8):1025-1033. https://doi.org/10.1097/ACM.0000000000000734
29. Bowen DJ, Kreuter M, Spring B, et al. How we design feasibility studies. *Am J Prev Med*. 2009;36(5):452-457. https://doi.org/10.1016/j.amepre.2009.02.002
30. Rickards G, Magee C, Artino AR Jr. You can't fix by analysis what you've spoiled by design: developing survey instruments and collecting validity evidence. *J Grad Med Educ*. 2012;4(4):407-410. https://doi.org/10.4300/jgme-d-12-00239.1

Received: September 1, 2021
Accepted: May 23, 2022
Published: July 12, 2022

Copyright © 2022 Goldman et al. This is an open-access publication distributed under the terms of the Creative Commons Attribution license.
